# Acute effects of NIV on peripheral muscle function and aerobic performance in patients with chronic obstructive pulmonary disease: a pilot study

**DOI:** 10.1186/s12890-022-02201-w

**Published:** 2022-11-04

**Authors:** Mariana Galvão de Medeiros Nogueira, Gabriely Azevêdo Gonçalo Silva, Marcelo Henrique Tavares Marinho, Ozana de Fátima Costa Brito, Wouber Hérickson de Brito Vieira, Marcela Abbott Galvão Ururahy, Ivan Daniel Bezerra Nogueira, Ivanízia Soares da Silva, Patrícia Angélica de Miranda Silva Nogueira

**Affiliations:** 1 Physiotherapy, Federal University of Rio Grande Do Norte, Av. Senador Salgado Filho, 3000, Candelária, Caixa Postal: 1524, Natal, Rio Grande Do Norte 59072970 Brazil; 2 Health Education, Federal University of Rio Grande Do Norte, Natal, Rio Grande Do Norte Brazil; 3 Department of Clinical and Toxicological Analysis, Federal University of Rio Grande Do Norte, Natal, Rio Grande Do Norte Brazil

**Keywords:** COPD, Skeletal muscle, Non-Invasive Ventilation

## Abstract

**Background:**

Non-invasive ventilation (NIV) reduces respiratory load and demands on peripheral muscles.

**Methods:**

This study aims to evaluate the acute effects of bi-level NIV on peripheral muscle function during isokinetic exercise and aerobic performance in chronic obstructive pulmonary disease (COPD) patients. This is a pilot crossover study performed with a non-probabilistic sample of 14 moderate to very severe COPD patients. Procedures carried out in two days. Dyspnea, quality of life, lung function, respiratory muscle strength, functional capacity (6-min walk test—6MWT), and isokinetic assessment of the quadriceps were assessed. Blood samples (lactate, lactate dehydrogenase, and creatine kinase concentration) were also collected. Right after, NIV was performed for 30 min (bi-level or placebo, according to randomization) followed by new blood sample collection, 6MWT, and isokinetic dynamometer tests. Before and after evaluations, the subjective perception of dyspnea and fatigue in the lower limbs was quantified. After a wash-out period of seven days, participants returned, and all assessments were performed again.

**Results:**

NIV showed improvements in perceived exertion and dyspnea after isokinetic exercise (*p* < 0.02 and *p* < 0.05, respectively).

**Conclusions:**

NIV improves the perception of dyspnea and fatigue during the isokinetic exercise.

## Introduction

Chronic obstructive pulmonary disease (COPD) is the third leading cause of death worldwide [1, 2] and will become the second most common cause of death in 2030 [3]. The natural history of the disease is associated with systemic manifestations (i.e., weight loss, nutritional abnormalities, and musculoskeletal dysfunction) contributing to functional decline [4]. Specifically, musculoskeletal dysfunction is characterized by sarcopenia and abnormal cell function leading to loss of strength and contributing to exercise intolerance, poor health status [1], and reduced health-related quality of life and survival [5].

Lower limb muscle weakness may be greater in moderate to severe COPD [6, 7], especially in the quadriceps femoris (QF), in which a considerable shift from type I to type II fibers occurs. When muscle fibers are metabolically depleted after acute effort, cell membrane permeability changes and elevates both muscle fatigue (lactate and lactate dehydrogenase [LDH]) and muscle damage (creatine kinase [CK]) [8] markers in the next 24 h. Expiratory flow limitation and increased respiratory rate reduce expiratory time, resulting in dynamic hyperinflation [9–11] associated with dyspnea and exercise intolerance and affecting functional capacity and quality of life [12].

Although non-invasive ventilation (NIV) has been proposed as a strategy to reduce exercise tolerance, dynamic hyperinflation, and dyspnea in COPD patients, the mechanisms underlying these improvements and the effects on LDH and CK serum levels are not fully understood. Thus, this study aimed to evaluate the acute effects of NIV on QF muscle function, functional capacity, and biochemical changes (i.e., lactate, LDH, and CK) in COPD patients. We hypothesized that NIV applied before isokinetic exercise increases exercise tolerance and improves peripheral lactate accumulation.

## Material and methods

### Study design and participants

This is a pilot randomized clinical trial study with a crossover design carried out at the Department of Physiotherapy of the Federal University of Rio Grande do Norte. The study protocol was approved by the *Research Ethics Committee of the Federal University of Rio Grande do* *Norte* (number 2,535,474) and conducted according to the resolution 466/12 of the National Health Council and the Declaration of Helsinki. All individuals involved signed an informed consent form.

Volunteers were recruited from the pulmonology service of a teaching clinic, as well as from basic health units, in the city of Natal/Rio Grande do Norte (RN). The randomization and order of application of NIV (BILEVEL or placebo) was defined using opaque envelopes containing the numbers 1 (BILEVEL) and 2 (PLACEBO), made by evaluator 1, who was instructed not to communicate this information to the volunteers and others. evaluators involved in the study.

Clinically stable COPD patients (diagnosed according to the Global Initiative for Obstructive Lung Disease) [1], of both sexes, without exacerbation or changes in medication for at least one month before the study, and no dyspnea during daily activities (grades 2, 3, and 4 of the Medical Research Council—MRC scale) [13] were included. Those with a diagnosis of neoplasia, orthopedic or neurological conditions affecting exercise capacity, peripheral arterial obstructive disease, heart failure, kidney or liver disease, did not adapt, or failed to complete the study procedures, were excluded.

### Instruments

#### Lung function and respiratory muscle strength

Lung function was assessed using a spirometer (Koko Digidoser, Longmont, USA), according to the ATS/ERS recommendations [14]. At least three tests were performed, with a variation of less than 5%, and the highest value obtained was compared with predicted values for the Brazilian population [15]. Forced vital capacity, forced expiratory volume in the first second, and FEV_1_/FVC ratio were evaluated.

Respiratory muscle strength was assessed using a digital manometer (MVD 300—Brazil). Maximum inspiratory and expiratory pressures were performed from residual volume and total lung capacity, respectively, and values were compared with previously reference values for the Brazilian population [16].

### Functional capacity

The 6-minute walk test (6MWT) was performed in a flat 30-m corridor, according to the ATS recommendations [17]. Subjects were instructed to keep walking for six minutes, and standardized verbal incentives were given each minute. The perception of dyspnea (using the 10-point Borg scale) were assessed at rest, at the end of the 6MWT, and two minutes after the test. In this test, the walked distance (in meters) was considered for analysis.

### Quadriceps femoris performance

The analysis of QF performance was performed in the dominant limb using a calibrated isokinetic dynamometer (Biodex Multi-Joint System 3 pro, USA). The same examiner performed all tests, and standardized verbal encouragement and instructions were strictly applied to all participants [18].

Subjects were instructed to remain seated, and the axis of rotation of the dynamometer arm was adjusted to the knee of the dominant limb [19]. All subjects were properly stabilized using belts attached to the thorax, hips, and thigh of the evaluated limb to avoid compensations. After weighting the limb, a familiarization session was conducted with the subject performing three knee flexion/extension repetitions at an angular speed of 240°/s. After two minutes of rest, concentric QF strength was evaluated with subjects performing 20 repetitions at the same speed. The following parameters were recorded: absolute and predicted peak torque values (PT and PT%, respectively), total work (W_T_), fatigue index (FI), and power (P). The protocol established was designed to reach a fatigue threshold in the QF muscle. Dyspnea and perceived level of exertion (Borg scale) were also assessed before and after isokinetic evaluation.

### Biochemical analyzes

Before and after procedures, venous blood samples were collected by a blinded experienced pharmacist to analyze lactate, LDH enzyme, and CK concentration. Samples were processed, centrifuged at 3000 rpm for 15 min at room temperature. The serum concentration was assessed using a specific enzyme kit for each biomarker, following manufacturer's recommendations.

### Quality of life and disease severity

The Portuguese version of the COPD Assessment Test (CAT) questionnaire was used to assess the quality of life. Subjects were instructed to choose only one option in each item of the questionnaire (cough, phlegm, chest tightness, breathlessness, limited activities, confidence leaving home, sleeplessness, and energy). Item scores range from 0 to 5 points resulting in a total score ranging from 0 to 40 points. The clinical impact of COPD was assessed according to the following stratification: mild (6–10), moderate (11–20), severe (21–30), and very severe (31–40) [20].

Dyspnea was assessed using the Medical Research Council (MRC scale) [13]. The scale allowed the volunteer to indicate the extent to which shortness of breath affected mobility during daily activities. The scores ranged from 1 to 5, with higher values indicating greater dyspnea.

COPD severity was assessed using the BODE index (body mass index [B], degree of airway obstruction [O], dyspnea [D], and exercise capacity [E]). For each variable, the following scores were assigned: body mass index (BMI), from 0 to 1 point; degree of airflow obstruction (FEV_1_% predicted), from 0 to 3 points; dyspnea (MRC scale), from 0 to 3 points; exercise capacity (walked distance in the 6MWT), from 0 to 3 points. The final score of the index ranges from 0 to 10, with higher scores indicating greater disease severity [21].

### Study design

**Fig. 1:**
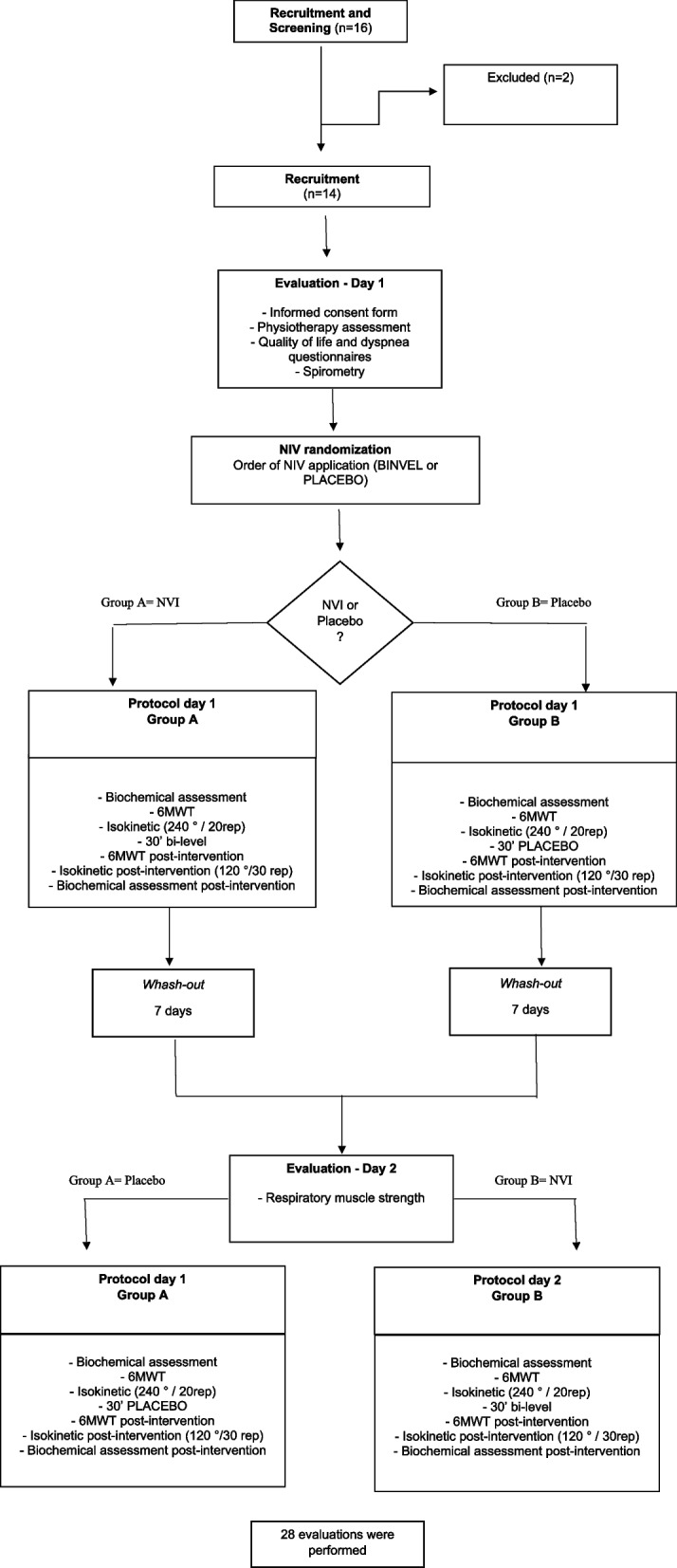
Flowchart of study design

Procedures were performed on two different days (performed seven days apart). On the first day, anthropometric data, clinical status, lung function, respiratory muscle strength, dyspnea (MRC scale), quality of life, disease severity, 6MWT, and isokinetic assessment were performed (Fig. 1).

Immediately after, subjects were randomized (simple drawing) to bi-level or placebo intervention. Both interventions were performed using a non-invasive (NIV) bi-level positive airway pressure device (Pro BiFlex, Philips Respironics, USA) and a nasal mask. First, the study participants were familiarized with the NIV and its interface for 5 min, and the appropriate level of positive pressure was selected, according to comfort and tolerance. After familiarization, 30 min of NIV [22] (bi-level mode or placebo, according to randomization) were performed. For the NIV intervention, two levels of positive airway pressure (bi-level) were used: the positive inspiratory airway pressure (IPAP) was fixed at 6 cmH_2_O, which could be gradually increased up to 14 cmH2O (2 cmH2O every minute), depending on the patient's comfort.While the expiratory airway pressure (EPAP) was set at 3 cmH2O, it could be gradually increased up to 7 cmH2O (1 cmH2O every minute), depending on the patient's comfort [22]. The same pressure chosen by the subject during adaptation was used in the placebo intervention; however, a T tube was connected between the equipment and the circuit, creating a leak and leaving the circuit open. The tube characteristics were: 22 × 18 × 22 mm nebulizer T-connector. The device's leakage compensation was 30L/min. The sensation of discomfort was measured using an arbitrary comfort score from 0 (no discomfort) to 5 (very uncomfortable). This parameter was used to limit the pressure increase and indicate the early interruption of the adaptation procedure, whenever the classification reached was 5. During both interventions, a researcher supervised and instructed the patient to perform nasal breathing with the mouth closed to avoid pressure leakage. Immediately afterward, the 6MWT, isokinetic assessment, and blood sample collection were conducted.

There was a wash-out period of seven days to ensure no interference from the previous intervention. Then, the participants were invited to return, and the same procedures were performed but with different NIV intervention, according to randomization.

The study was performed by three investigators. One was aware of the NIV intervention, another collected physiological data, while the third operated the dynamometer to use the same verbal command intensity.

### Statistical analysis

Sample size was defined based on the PT variable of a previous study [22], acquired before and after NIV intervention. Adopting a significance level of 5% and a study power of 80%, the optimal number was estimated as 14 individuals.

Data are shown as mean ± standard deviation, and 95% confidence intervals. Data normality was performed using the Shapiro–Wilk test. Normal Data was available for repeated measures analysis of variance (ANOVA) using post-test Bonferroni. Data with non-normal distribution we used the Friedman test followed by the Wilcoxon post hoc test. Descriptive and inferential analyses were conducted using the SPSS software, version 20 statistical (IBM Corp. USA). For all statistical analyzes, a significance level of  < 0.05 (two-tailed) was adopted.

## Results

Sixteen subjects were included, but two were excluded for not showing up for the second evaluation after the wash-out time. Thus, the sample was composed of 14 COPD subjects (10 female) with mean age of 62.8 ± 8.8 years and BMI in the overweight range (28.4 ± 3.8). Twelve participants reported using approximately 375.4 pack/years, and COPD was classified as severe in 57% and moderate in 43% of the sample [1]. Mean BODE index was 8.1 ± 1.6, and a moderate clinical impact on the quality of life was found (mean CAT value of 18 ± 9.9). Mean IPAP and EPAP values were 13.71 ± 0.72 and 7.85 ± 0.36 cmH_2_O, respectively. The results of dyspnea and lower limb effort in the 6MWT and isokinetic evaluation after NIV or placebo are shown in Table 1.

**Table 1:** Dyspnea and lower limb effort in the 6MWT and isokinetic evaluation after NIV or placebo

	**NIV**				**Placebo**				**Intergroup** **Post NIV and Post placebo**	
	**Pre**	**Post**	***P* value**	**95%CI**	**Pre**	**Post**	**P value**	**95%CI**	***P* value**	**95%CI**
Lower limb effort – 6MWT	11.07 ± 3.02	6.86 ± 2.14	0.00	(-6.72 to -1.71)	12.64 ± 3.20	11.43 ± 3.78	1.00	(-4.74 to 2.31)	0.01	(-7.94 to -1.21)
Lower limb effort – iso	10.19 ± 3.78	15.36 ± 2.34	0.00	(1.45 to 8.89)	11.43 ± 2.56	12.21 ± 3.47	1.00	(-1.98 to 3.55)	0.06	(-0.11 to 6.40)
Dyspnea – 6MWT	4.25 ± 2.91	3.25 ± 3.05	0.97	(-3.09 to 1.09)	4.50 ± 2.28	4.71 ± 3.00	1.00	(-1.01 to 1.44)	0.67	(-4.13 to 1.20)
Dyspnea – iso	2.79 ± 2.32	4.50 ± 2.90	0.03	(0.15 to 3.28)	2.25 ± 2.17	5.14 ± 2.38	0.23	(-0.66 to 4.45)	0.23	(-4.45 to 0.66)

*6MWT* 6-minute walk test, *NIV* Noninvasive ventilation, *Iso* Isokinetic evaluation

As shown in Table 1, NIV led to a mean lower limb effort reduction of 4.6 after isokinetic assessment compared with placebo (*p* = 0.02). Dyspnea was also lower in NIV group compared with placebo (*p* = 0.05). Table 2 shows the QF performance during the isokinetic evaluation. Although statistically significant differences were observed after NIV and placebo, no intergroup differences were observed after interventions. Interventions were also not able to increase performance in the 6MWT.

**Table 2:** QF isokinetic performance

	**NIV**				**Placebo**				**Intergroup** **Post NIV and Post placebo**	
	**Pre**	**Post**	***P* value**	**95%CI**	**Pre**	**Post**	***P* value**	**95%CI**	***P* value**	**95%CI**
PT (Nm)	42.01 ± 15.74	57.68 ± 17.71	0.00	7.79 to 23.55)	47.26 ± 14.95	59.35 ± 24.26	0.21	(-398 to 28.15)	1.00	(10.60 to 7.26
PT% (%)	40.68 ± 26.19	48.67 ± 31.39	0.20	(-9.73 to 25.70)	43.90 ± 29.00	52.22 ± 40.32	0.90	(-14.95 to 31.58)	1.00	(-26.08 to 18.99)
W_T_ (J)	740.10 ± 250.16	1209.95 ± 328.52	0.00	(266.85 to 672.86)	849.66 ± 260.30	1233.26 ± 531.29	0.08	(-34.93 to 802.13)	1.00	(-318.94 to 272.32)
FI (%)	0.55 ± 23.82	34.67 ± 10.14	0.00	(18.78 to 49.46)	24.45 ± 19.81	26.63 ± 36.37	1.00	(-33.53 to 37.89)	1.00	(-23.04 to 39.12)
P (W)	76.08 ± 22.40	62.90 ± 18.16	0.15	(-29.35 to 2.98)	88.71 ± 29.67	66.55 ± 20.85	0.01	(-38.15 to -6.17)	1.00	(-16.58 to 9.29)

*NIV* Noninvasive ventilation, *PT* Peak torque, *PT%* Predicted peak torque, *W*_*T*_ Total work, *IF* Fatigue index, *P* Power, *Nm* Newton meter, *%* Percentage, *J* Joule, *W* Watts

Only lactate concentration was significantly different after both interventions (*p* < 0.001) (Table 3). However, no differences were observed between interventions.

**Table 3:** Blood lactate, LDH and CK levels

	**NIV**				**Placebo**				**Intergroup** **Post NIV and Post placebo**	
	**Pre**	**Post**	***P* value**	**95%CI**	**Pre**	**Post**	***P* value**	**95%CI**	***P* value**	**95%CI**
CK	141.82 ± 129,02	141.91 ± 132.99	0.13	(-19.03 to 19.21)	127.27 ± 117.58	126.18 ± 112.49	0.30	(-16.31 to 14.13)	0.21	(-38.77 to 70.23)
LDH	411.36 ± 89.08	417.91 ± 140.74	1.00	(-80.74 to 93.84)	444.82 ± 56.23	406.18 ± 112.52	1.00	(-143.65 to 66.38)	1.00	(-58.37 to 81.83)
Lactate	20.00 ± 10.91	31.73 ± 11.16	0.00*	(4.55 to 18.91)	16.82 ± 5.10	29.91 ± 10.93	0.01*	(3.13 to 23.05)	1.00	(-11.55 to 15.18)

*CK* Creatine, *LDH* Lactate dehydrogenase

* Statistically significant

## Discussion

The present study was designed to evaluate the acute effects of non-invasive bi-level positive airway pressure on peripheral muscle function (quadriceps femoris), biochemical changes, and functional capacity of COPD patients. Fourteen participants were evaluated, 57% with severe COPD. The main finding was that NIV increased perceived exertion in the lower limbs and dyspnea during isokinetic evaluation.

A significant reduction of 4.6 points in lower limb fatigue during isokinetic exercise was observed between groups. Peripheral muscle fatigue is rarely the primary outcome evaluated in large clinical trials; therefore, studies evaluating fatigue in COPD patients are scarce [23]. However, COPD patients increase respiratory work during exercise and deviate blood flow to respiratory muscles to optimize breathing, reducing peripheral blood flow and causing early fatigue due to peripheral blood restriction [23].

The reduced perception of fatigue may be due to respiratory comfort provided by NIV, reducing respiratory muscle overload, optimizing pulmonary mechanics, and allowing effective blood flow to the periphery [24]. Also, NIV can improve dyspnea and exercise capacity, allowing moderate to severe COPD patients to exercise at higher intensities, increasing endurance and the distance covered.

Dyspnea during isokinetic exercise was significantly different between groups after intervention. Thus, it is believed that NIV improves exercise tolerance in COPD patients due to an immediate improvement in dyspnea and airway resistance since it reduces dynamic hyperinflation and respiratory work [22, 25].

No significant difference was observed in lower limb fatigue and dyspnea during the 6MWT, probably because the 6MWT requires a more physiological demand associated with the ADL performance, allowing adaptation to this submaximal test.

According to the first study evaluating NIV effects on QF during concentric exercise in severe COPD patients, ventilatory aid reduced FI of this muscle [22], differing from our results. These results are mainly due to study design differences since NIV was applied before exercise in our study, while Borghi et al. [22] performed it concomitantly with exercise. These authors also used two different groups, unlike the current study that used the same patients on different days. In this sense, a learning effect cannot be ruled out since patients performed two days of evaluation, which may have influenced the results of the placebo group.

No significant difference was found in the distance covered, contrary to studies demonstrating improvements in the NIV group. Intragroup differences after NIV demonstrated increased distance covered (~ 16 m), approaching a clinically significant difference of 25 meters [26]. We believe that the small number of volunteers, the fact that 6MWT is a submaximal test, the learning effect, and reduced NIV application time may have limited the relevance of this data. However, a 40-m improvement in the 6MWT was observed in 15 COPD patients submitted to nocturnal NIV, probably related to increased peripheral muscle strength [27]. In the same study, patients improved distance walked in 83 m after NIV and pulmonary rehabilitation program. Most studies showed benefits of NIV therapy during physical exercise [24, 26, 27]. Currently, studies applying NIV before exercise are scarce and evidence regarding the duration of the NIV effects.

Strategies to reduce the respiratory muscle workload tend to be accompanied by reduced peripheral muscle fatigue. These findings may be based on NIV benefits, such as increased functional residual capacity with expanded alveoli maintenance and consequent increase in ventilatory reserve and reduced ventilatory workload [28]. It may be considered that the lower the ventilatory work, the lower the cardiac output, reducing sympathetic response of the peripheral muscles. Consequently, this increases blood and oxygen supply to these areas, improving exercise tolerance [29].

Regarding biochemical variables, no significant differences between the NIV group and placebo were observed. It is possible that the protocol used in the present study was not sufficient to generate significant muscle damage to increase biochemical markers levels. Also, these markers were evaluated immediately after stress, hindering the assessment of peak serum levels since they are observed after 24 to 48 hours [30].

Studies regarding the effects of eccentric exercise on muscle damage markers in moderate to severe COPD patients have shown that submaximal eccentric exercise leads to significantly more muscle damage than healthy controls. Eccentric contractions contribute to greater muscle damage compared with concentric contractions [31].

Blood lactate was also not different between groups. By improving oxidative capacity, COPD patients might increase exercise workload with less lactate production. However, NIV did not reduce lactate levels, probably because NIV was not provided during exercise, and time was not adequate to detect possible lactate changes. The type of exercise may have also interfered with NIV benefits, considering that the isokinetic protocol preferentially uses anaerobic metabolism [32].

Short-term NIV in COPD patients reduces diaphragm overload in upper and lower limb exercises, accompanied by an improvement in blood flow to the muscles, mainly of the lower limbs, reducing the competition of respiratory and peripheral muscle blood flow. The values ​​and time of use of NIV based on the protocol by Borghi (2009) may not have been sufficient to achieve the expected benefits. Data from one study show that the mean IPAP and EPAP used for COPD patients was 16.5 ± 4.7 cmH_2_O and 4.7 ± 4.7 cmH_2_O, respectively. The corresponding values ​​after three months were 17.9 ± 5.2 cmH_2_O and 4.6 ± 1.4 cmH_2_O, respectively, for improved distance on the 6MWT. After 12 months, the mean IPAP was 16.8 ± 5.4 cmH 2 O and the mean EPAP was 4.9 ± 1.5 cmH_2_O. Chronic NIV was used for an average of 6.2 ± 2 0.8 h after three months and 5.5 ± 3.2 h after 12 months. These 3- and 12-month follow-up values ​​improved distance at the 6MWT [33].

## Conclusion

New studies with different isokinetic and/or aerobic protocols should be used to select the most appropriate proposal for COPD, together with a more comprehensive physical training program to improve physical performance in this population.

This study is not free of limitations. Sample size and study design should be considered when applying the results to the broader COPD population, as well as NIV during isokinetic exercise and 6MWT to obtain significant effects.

## Data Availability

All requests for raw and analyzed data and materials can be found at: https://doi.org/10.5281/zenodo.6478448.

## Abbreviations

NIV: Non-invasive ventilation
COPD: Chronic obstructive pulmonary disease
6MWT: 6-Minute walk test
QF: Quadriceps femoris
LDH: Lactate dehydrogenase
CK: Creatine kinase
MRC: Medical research council
VEF1: Forced expiratory volume in the first second
PT: Absolute peak torque values
PT%: Predicted peak torque values
Wt: Total work
FI: Fatigue index
P: Power
CAT: COPD Assessment Test
BODE: Mass index, airway Obstruction, Dyspnea, and Exercise capacity
BMI: Body mass index
IPAP: Positive inspiratory airway pressure
EPAP: Expiratory airway pressure
